# Investigation of the Effect of Stress on Oxygen Diffusion in Pure Titanium Using a Phase-Field Model

**DOI:** 10.3390/ma17071539

**Published:** 2024-03-28

**Authors:** Yaomian Wang, Mengqi Zhang, Huanping Yang

**Affiliations:** School of Metallurgical Engineering, Xi’an University of Architecture and Technology, Xi’an 710055, China; zhangmengqi113@163.com

**Keywords:** diffusion, titanium, simulation

## Abstract

Diffusion plays a vital role during the fabrication of many materials. It is a well-known fact that stress can influence diffusion behavior. In order to optimize material processing techniques, a quantitative evaluation of the effect of stress on diffusion is essentially required. By analyzing the free energy change in a Ti-O system during diffusion, a phase-field model was developed to address this issue. Using this model, the diffusion of oxygen atoms in pure titanium under different stress states was investigated. It was observed that the true equilibrium concentration of oxygen was proportional to its hydrostatic pressure. Tensile stress can increase the oxygen concentration. This raise in concentration decreased with temperature. However, the promotion of diffusion can be attained in deeper regions at a higher temperature. On the contrary, compressive stress inhibited the diffusion of oxygen in pure titanium. Under a certain compressive stress, the decrease in the oxygen concentration at the surface layer was more significant at a lower temperature, while a decrease could be observed at a deeper distance from the surface at a higher temperature. A thermodynamic explanation of the effect of stress on diffusion was given based on the proposed phase-field model.

## 1. Introduction

Diffusion is a fundamental phenomenon that occurs in many material processing techniques. It usually refers to the process of the movement of particles, molecules, atoms, or ions from a region of a higher concentration to a region of a lower concentration. This movement occurs due to the random thermal motion of particles and is driven by the tendency of system to achieve thermodynamic equilibrium. Diffusion plays a critical role in several important processes, such as heat treatment [1], hot working [2], solid-state phase transformations [3], powder metallurgy [4], welding [5], and thermochemical treatment [6,7]. During these processes, diffusion affects the evolution of the microstructure of and mechanical properties of materials.

The diffusion rate is influenced by a variety of factors, including the lattice structure of the material, the type of diffusing species, the interaction between the diffusing species and the matrix, the concentration gradient, and temperature. Moreover, the stress state, which might be inner stress resulting from plastic deformation or externally applied stress, may also significantly affect the diffusion behavior. Research has shown that the residual stress introduced by shot peening can slow the diffusion of oxygen in zirconium [8]. Experiments on P92 steel also revealed that the compressive residual stress caused by shot peening reduced oxygen diffusion [9]. It has been reported that in the carburization of 316 austenitic stainless steel, compressive stress retarded the diffusion of carbon atoms from the carburized surface towards the central substrate compared to without stress [10]. Experiments on a compact tension-type specimen of 20% cold-worked-type 316 stainless steel indicated that the diffusion coefficient of nickel at 450 °C under a tensile stress of 553 MPa was 6.5 times higher than that of a sample without tensile stress [11]. In the DZ125 Ni-based superalloy, under a tensile stress of 100 MPa, it was found that the diffusion rate of Cr was improved compared to in unstressed samples [12].

As an important engineering material, pure titanium has been extensively utilized in some fields, such as biomedical devices, aerospace, and the petrochemical industry [13,14,15]. However, its hardness and wear resistance are low, which limits its use. Various processing methods, including severe plastic deformation [16,17,18], thermal oxidation [19,20,21], carburization [22,23,24], nitriding [7,25,26], and boriding [7,27,28], have been proposed to improve its mechanical properties. During the processes of thermal oxidation, carburization, nitriding, and boriding, the atom diffusion behavior in pure titanium plays a key role in achieving good mechanical properties. Attempts have been made to promote oxygen diffusion in pure titanium by applying an external bending strain [29]. It has been reported that tensile strain promotes the diffusion of oxygen. However, the effect of compressive strain on the diffusion process was not obvious. In order to achieve an unambiguous understanding of stress’s effect on oxygen diffusion in pure titanium and optimize the processing method for property optimization, it is necessary to explore this issue in more detail.

In the ordinary way, diffusion behavior is described using Fick’s law or its modified form. Nevertheless, a different method is proposed to address this issue in the present study. Based on analyzing the free energy change during oxygen diffusion into titanium under stress, we developed a phase-field model to illustrate the effect of stress on the oxygen diffusion behavior in titanium. Comparisons with Fick’s law and another model were made to verify the present phase-field model. Applying this model, the influence of stress on the diffusion was investigated and then discussed theoretically.

## 2. Model Description

### 2.1. Chemical Energy

Oxygen is substantially soluble in titanium over a wide temperature range. It can be up to 14% by weight, which is equal to 33% by mole fraction [30,31]. A change in the oxygen concentration causes variations in the chemical energy of the Ti-O solution system. In the present study, the chemical energy $E_{mol}$ is illustrated as an increment relative to the equilibrium state, which is expressed as:

(1) $$E_{mol}=G_{mol}-G_{e}$$

where $G_{mol}$ is the Gibbs free energy of the solution for one mole and $G_{e}$ is the corresponding Gibbs free energy at equilibrium. The value of $E_{mol}$ increases as the oxygen concentration deviates from the equilibrium.

An ideal solution model was used to estimate the molar Gibbs free energy $G_{mol}$ of the solution, which is given by:

(2) $$G_{mol}=\left(1-c\right)\mu_{A}^{0}+c\mu_{B}^{0}+RT(\left(1-c\right)\ln\left(1-c\right)+clnc)$$

where c is the mole fraction of the solute atoms, R is the gas constant, T is the absolute temperature, and $\mu_{A}^{0}$ and $\mu_{B}^{0}$ are the chemical potentials of the solvent and solute, respectively. Neglecting the effect of atmospheric pressure, $\mu_{A}^{0}$ and $\mu_{B}^{0}$ are functions of temperature only. When $\partial G_{mol}/\partial c=0$, the equilibrium concentration $c_{e}$ at different temperatures can be determined. The corresponding value of $\left(\mu_{A}^{0}-\mu_{B}^{0}\right)$ can be calculated through fitting using the Ti-O phase diagram.

Then, $E_{mol}$ can be calculated as follows:

(3) $$E_{mol}=\left(c-c_{e}\right)\left(\mu_{B}^{0}-\mu_{A}^{0}\right)+RT[\left(1-c\right)\ln\left(1-c\right)+clnc-\left(1-c_{e}\right)\ln\left(1-c_{e}\right)-c_{e}lnc_{e}]$$

**Figure 1:**
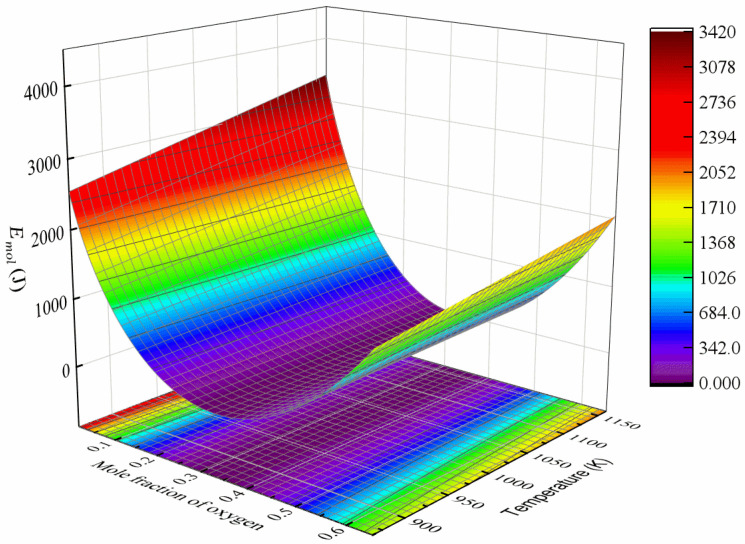
Chemical energy of one-mole Ti-O solution system as function of oxygen mole fraction and temperature.

The chemical energy computed using Equation (3) is shown in Figure 1. It can be seen that at a constant temperature, the chemical energy reaches its minimum value as the oxygen concentration achieves its equilibrium. By increasing the temperature, the chemical energy increases for the alloy with a non-equilibrium oxygen concentration.

**Figure 2:**
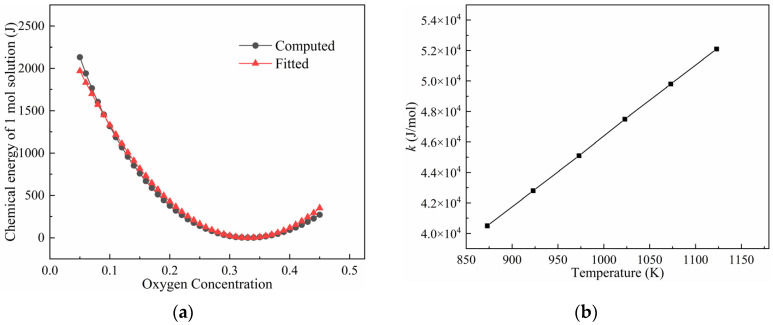
(**a**) Comparison of the computed energy and fitted chemical energy of one-mole Ti-O solution at temperatures of 1073 K and (**b**) the variation in coefficient k with temperature.

Using series expansion of the natural logarithm and ignoring the high-order terms, the chemical energy $E_{mol}$ given using Equation (3) can be expressed approximately using a second-order polynomial. Following the work by Echebarria et al. [32] and Zaeem et al. [33], it is rewritten in the form:

(4) $$E_{mol}=\frac{1}{2}k{\left(c-c_{e}\right)}^{2}$$

where k is the coefficient. The chemical energies computed using Equation (3) are fitted using Equation (4) for the Ti-O solution system at various temperatures, and the results are shown in Figure 2. It is evident that the fitted results are in good agreement with those computed based on thermodynamic theories. The coefficient k increases with an increase in temperature, which indicates that the system can achieve a higher chemical energy at an elevated temperature.

Within a unit volume, the chemical energy E is given by:

(5) $$E=E_{mol}\rho /m_{mol}$$

where ρ is the density of the solution and $m_{mol}$ is the molar mass.

### 2.2. Work Undertaken on the Solute Atoms and the True Equilibrium Concentration

In the present model, a stress field with normal stresses $\sigma_{xx}$, $\sigma_{yy}$, and $\sigma_{zz}$ is considered. These stresses may be externally applied stresses or inner stresses. Assuming that a spherical solute atom diffuses into the solvent metal, the work undertaken $w_{0}$ on the atom can be given by:

(6) $$w_{0}=\frac{4}{3}\pi r^{3}P$$

where r is the radius of the solute atom and P is the hydrostatic pressure, expressed as $P=(\sigma_{xx}+\sigma_{yy}+\sigma_{zz})/3$. As the solute concentration increases to c, the energy changes due to the work carried out for the one-mole Ti-O solution system are calculated as follows:

(7) $$W_{mol}=-w_{0}N_{A}c$$

where $N_{A}$ is the Avogadro constant.

**Figure 3:**
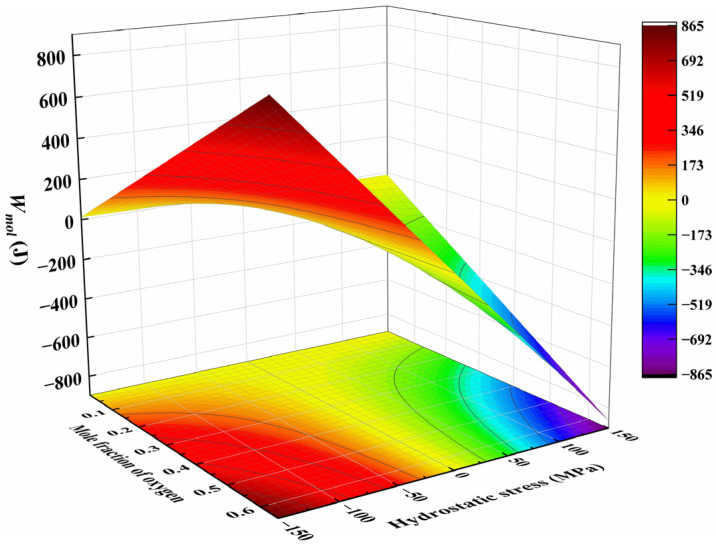
Energy change resulting from work undertaken in one-mole Ti-O solution system as a function of oxygen mole fraction and hydrostatic stress.

Figure 3 illustrates the variation in $W_{mol}$ with a mole fraction of oxygen and hydrostatic stress. The energy changes resulting from the work undertaken increase with an increase in the solute concentration and the magnitude of hydrostatic stress. Under tensile stress, the energy changes are negative, which indicates that tensile stress facilitates oxygen diffusion. On the contrary, compressive stress results in a positive energy change, which hinders oxygen diffusion.

Similarly, the work undertaken per unit volume, W, can be expressed as follows:

(8) $$W=W_{mol}\rho /m_{mol}$$

The stress considered in this model can be uniform or inhomogeneous. In addition, an updated hydrostatic pressure at a new time interval can be used to model the evolution of stress during diffusion. In this study, the influence of uniform tensile stress, compressive stress, and the stress gradient on diffusion was examined.

By combining the effects of the chemical energy E and the work undertaken W, the true equilibrium concentration $c_{e}^{t}$ affected by temperature and the stress state can be determined using the following condition:

(9) $$\frac{\partial (E+W)}{\partial c}=0$$

Then, we can obtain:

(10) $$c_{e}^{t}=c_{e}+\frac{4{\pi r}^{3}PN_{A}}{3k}$$

**Figure 4:**
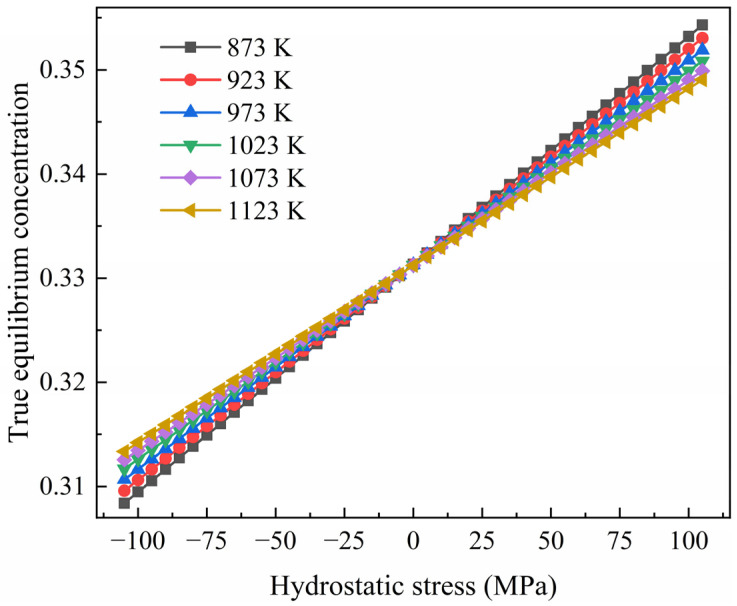
Variation in the true equilibrium concentration of oxygen in pure titanium with hydrostatic stress at different temperatures.

Here, $\frac{4{\pi r}^{3}PN_{A}}{3k}$ represents the contribution of stress to the true equilibrium concentration $c_{e}^{t}$. This contribution is directly proportional to stress and inversely proportional to the chemical energy coefficient, which is dependent upon temperature. The variation in the true equilibrium concentration $c_{e}^{t}$ with stress at different temperatures is shown in Figure 4. The results reveal that the true equilibrium concentration $c_{e}^{t}$ rises significantly as the stress increases from −100 MPa to 100 MPa.

### 2.3. Free Energy Functional and the Conserved Phase-Field Equation

The free energy change in the Ti-O solution system resulting from oxygen diffusion under stress consists of chemical energy based on its chemical composition and the work undertaken on the solute atoms due to stress. The free energy functional F can be written as:

(11) $$F=\int_{V}^{\;}(W+E)dv$$

where V is the total volume of the Ti-O solution system. This equation for the free energy functional is similar to the expression proposed by Zaeem et al. [33]. In their work, the free energy, which affects the oxygen diffusion, consists of the chemical energy and an elastic strain energy term resulting from the phase transformation.

In the phase-field framework, the evolution of the conserved variable in the direction that decreases the free energy of the entire system is determined by the governing equation [32,33,34]:

(12) $$\frac{\partial c}{\partial t}=\nabla \cdot \left(M\nabla \frac{\delta F}{\delta c}\right)$$

where $M=\frac{Dm_{mol}}{k\rho }$ is the mobility coefficient. The diffusion coefficient D is a temperature-dependent parameter, which is calculated using the expression $D=D_{0}exp(-Q/RT)$. Here, $D_{0}=4.08\times 10^{-5}\;m^{2}\cdot s^{-1}$ is the pre-exponent, and $Q=197\times 10^{3}\;J\cdot {\mathrm{mol}}^{-1}$ is the activation energy [35]. In this simulation, it is assumed that the diffusion coefficient remains constant with varying spatial positions. By taking a functional derivative of Equation (11), the dynamic Equation (12) becomes:

(13) $$\frac{\partial c}{\partial t}=M\nabla^{2}(\frac{\partial W}{\partial c}+\frac{\partial E}{\partial c})$$

It is well known that an oxide film can form on the surface of pure titanium at a high temperature. The influence of an oxide film on the diffusion was not considered in this study since the thickness of the oxide film is much smaller than the depth of diffusion. The computation was based on the assumption that the concentration achieves its equilibrium on the surface of pure titanium.

The influence of stress on diffusion can be evaluated by solving Equation (13). For a uniform stress field which is position-independent, the true equilibrium concentration is affected as described by Equation (10), though $\nabla^{2}\left(\frac{\partial W}{\partial c}\right)=0$. For an inhomogeneous stress field, where the stress varies in its coordinates, $\nabla^{2}\left(\frac{\partial W}{\partial c}\right)$ is determined by the spatial distribution of the stress. If the stress is zero, the terms $\frac{\partial W}{\partial c}$ in Equation (13) and $\frac{4{\pi r}^{3}PN_{A}}{3k}$ in Equation (10) vanish, and the present phase-field model is reduced to Fick’s second law of diffusion, which is expressed as:

(14) $$\frac{\partial c}{\partial t}=D\nabla^{2}c$$

The phase-field equation was solved using a finite difference method. The simulation domain was rectangular, with a grid size of 10 nm.

## 3. Results and Discussion

In this research, different stress fields were applied to pure titanium considering its mechanical properties at elevated temperatures [36]. The influence of uniform tensile stress, compressive stresses, and the stress gradient on the oxygen diffusion in pure titanium was explored using the phase-field model.

### 3.1. Influence of Tensile Stress

**Figure 5:**
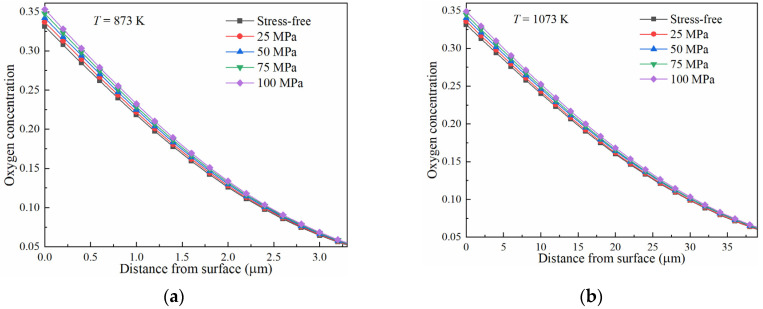
Influence of tensile stress on the diffusion of oxygen in pure titanium for 10 h at temperatures of (**a**) 873 K and (**b**) 1073 K.

The diffusion of oxygen in pure titanium was investigated by applying uniform tensile stress ranging from 0 MPa to 100 MPa. Different temperatures were considered in the simulation. The computed distributions of the oxygen concentration after diffusion at 873 K and 1073 K for 10 h are illustrated in Figure 5. It is evident that the oxygen concentration was increased by increasing the tensile stress at different temperatures. This indicates that tensile stress can promote the diffusion of oxygen atoms in pure titanium.

**Figure 6:**
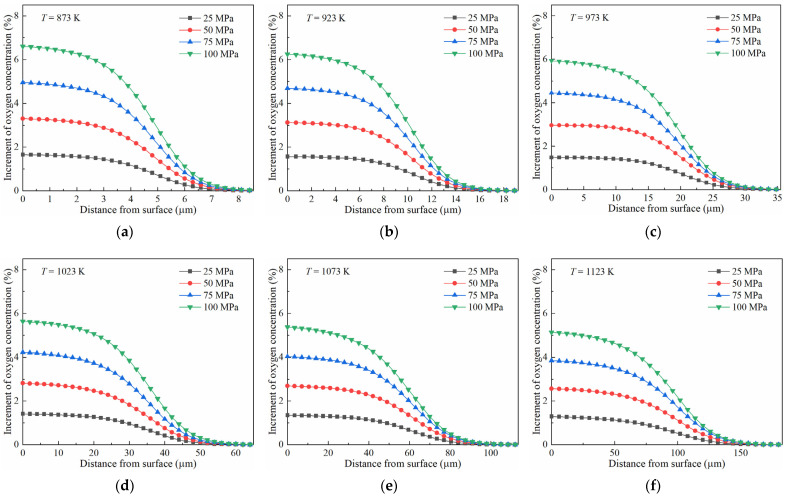
Increment in oxygen concentration in pure titanium under tensile stress compared to the stress-free state at different temperatures: (**a**) 873 K, (**b**) 923 K, (**c**) 973 K, (**d**) 1023 K, (**e**) 1073 K, and (**f**) 1123 K.

The increase in the oxygen concentration in the samples under tensile stress compared to in the stress-free state at different temperatures is calculated, and the results are depicted in Figure 6. It is observed that the increment in the oxygen concentration was about 7% in the surface layer of the samples diffused at 873 K under 100 MPa of tensile stress. The increment in the oxygen concentration decreases with an increase in the distance from surface. Moreover, the influence of stress on the oxygen diffusion varies with temperature. At a lower temperature, the increment is more prominent in the surface layer of the samples. At a high temperature of 1123 K, only a ~5% increment is observed in the surface layer of the samples. However, the stretched samples diffused at a high temperature could acquire a greater depth, which showed an increment in their oxygen concentration compared to the stress-free ones.

It has been demonstrated that the true equilibrium concentration increased with stress and decreased with temperature, which is illustrated in Figure 4. The oxygen concentration in the surface layer rapidly approached equilibrium during diffusion; therefore, the increment in the oxygen concentration increased with tensile stress, as shown in Figure 6. For the samples diffusing at a lower temperature and showing a higher increment in the oxygen concentration in their surface layers, this can also be attributed to their higher true equilibrium concentration.

**Figure 7:**
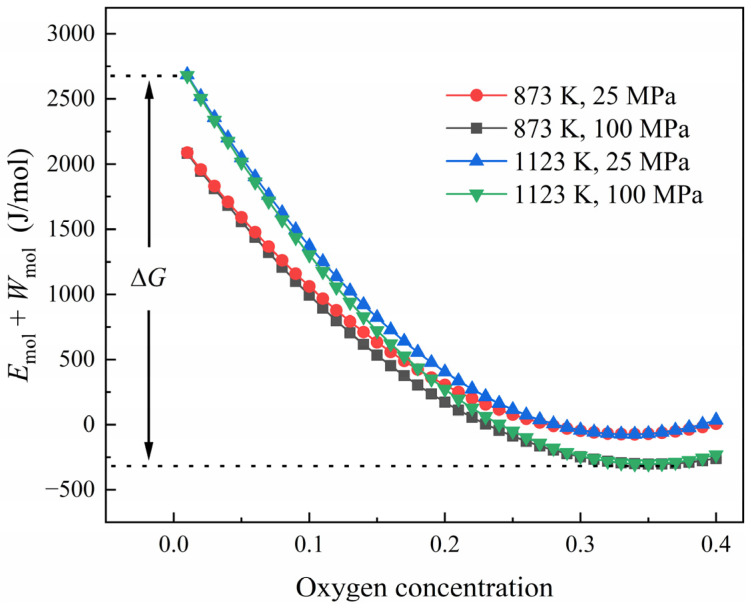
Change in free energy density with oxygen concentration.

The free energy density decreased as the oxygen concentration approached equilibrium. The change in the free energy ∆G varied with temperature and stress, which is shown in Figure 7. It can be observed that a higher temperature and tensile stress lead to a greater change in free energy. This indicates that the diffusion can be enhanced by applying a high tensile stress and a high temperature. For this reason, pure titanium diffused at 1123 K under 100 MPa of tensile stress, exhibiting the greatest distance with the promoted oxygen concentration in the simulation.

### 3.2. Influence of Compressive Stress

**Figure 8:**
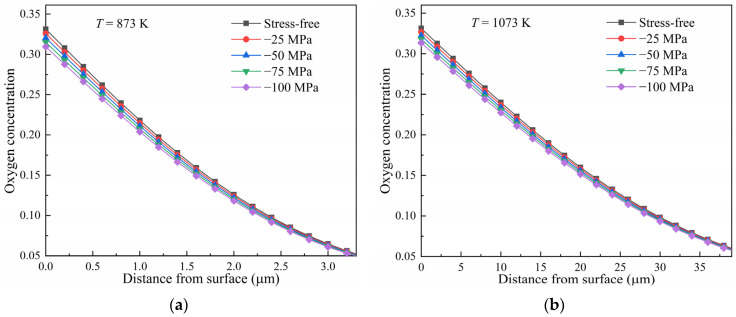
Influence of compressive stress on the diffusion of oxygen in pure titanium for 10 h at temperatures of (**a**) 873 K and (**b**) 1073 K.

**Figure 9:**
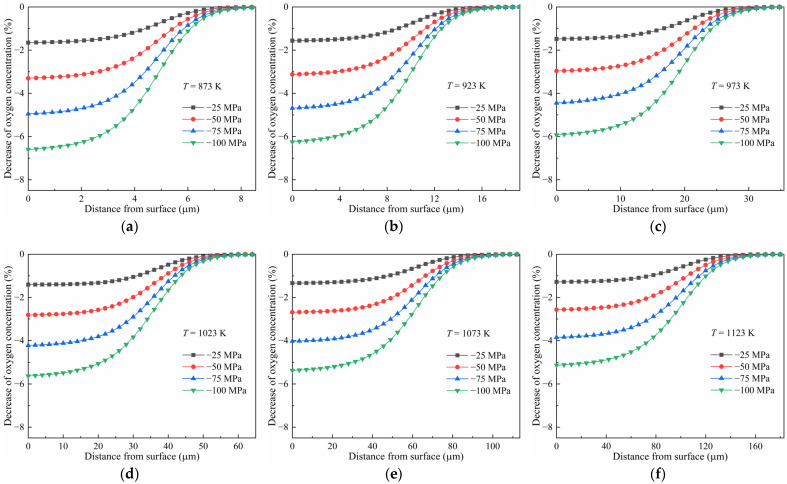
Decrease in oxygen concentration of pure titanium under compressive stress compared to the stress-free state at different temperatures: (**a**) 873 K, (**b**) 923 K, (**c**) 973 K, (**d**) 1023 K, (**e**) 1073 K, and (**f**) 1123 K.

The diffusion under uniform compressive stress at different temperatures was computed. The predictions after diffusion at 873 K and 1073 K for 10 h are shown in Figure 8. Contrary to tensile stress, compressive stress inhibits the diffusion of oxygen in pure titanium. The oxygen concentration of the samples under compressive stress is lower than that of the stress-free ones. The decrease in the oxygen concentration compared to in the stress-free state is shown in Figure 9. It is shown that the oxygen concentration decreased by about 2% in the surface layer for the titanium diffused at 873 K under a compressive stress of −25 MPa. The decrease reached about 7% under a compressive stress of −100 MPa, as shown in Figure 9a. Evidently, the decrease in concentration increased with compressive stress. However, it became less as the temperature was increased. At a temperature of 1123 K, the decrease reduced to about 5% under a compressive stress of −100 MPa.

The true equilibrium concentration of oxygen decreases with an increase in the compressive stress, as shown in Figure 4. This means that the difference between the initial concentration and the equilibrium became smaller with an increase in the compressive stress, thus reducing the driving force for oxygen diffusion. This is why there is a greater decrease in the oxygen concentration with an increase in compressive stress. Under compressive stress, the decrease in the true equilibrium concentration is less as the temperature is increased. Therefore, the decrease in the oxygen concentration in the samples diffused at a high temperature is smaller than that in those diffused at a lower temperature.

### 3.3. The Influence of the Stress Gradient

**Figure 10:**
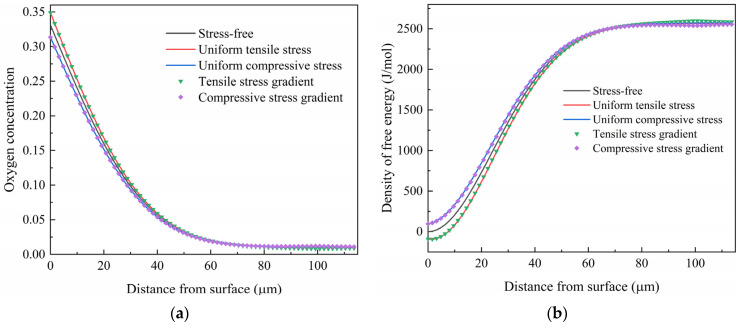
Influence of stress state on the distribution of (**a**) oxygen concentration and (**b**) density of free energy after diffusion at 1073 K for 10 h.

A tensile (compressive) stress varying linearly from 100 MPa (−100 MPa) at the surface to 0 MPa at a depth of 100 μm was used to examine the influence of the stress gradient on diffusion. The oxygen concentration distribution after diffusion at 1073 K for 10 h is illustrated in Figure 10a. The results obtained under a uniform tensile stress of 100 MPa and a compressive stress of −100 MPa, as well as in a stress-free state, are also given for comparison. It is shown that the oxygen concentration distribution of the titanium under gradually decreasing tensile stress is almost the same as that under uniform tensile stress, except at a depth of about 100 μm. Similarly, the gradually decreasing compressive stress and the uniform compressive stress also showed no obvious differences in their oxygen concentration distribution.

The corresponding distribution of the free energy density is shown in Figure 10b. A negative free energy density is obtained for the gradually decreased tensile stress and the uniform tensile stress in the surface layer at a depth of less than 10 μm. This can be attributed to the low chemical energy density of the surface layer and the work undertaken by the tensile stress. When increasing the depth, the oxygen concentration decreases, and thus the chemical energy density increases; therefore, the density of the free energy becomes positive. For the sample under uniform compressive stress and gradually decreasing compressive stress, the free energy density is higher because of the work carried out by the compressive stress. It is noted that the difference in density of free energy between the sample under uniform stress and the one under gradual decreased stress is little.

**Figure 11:**
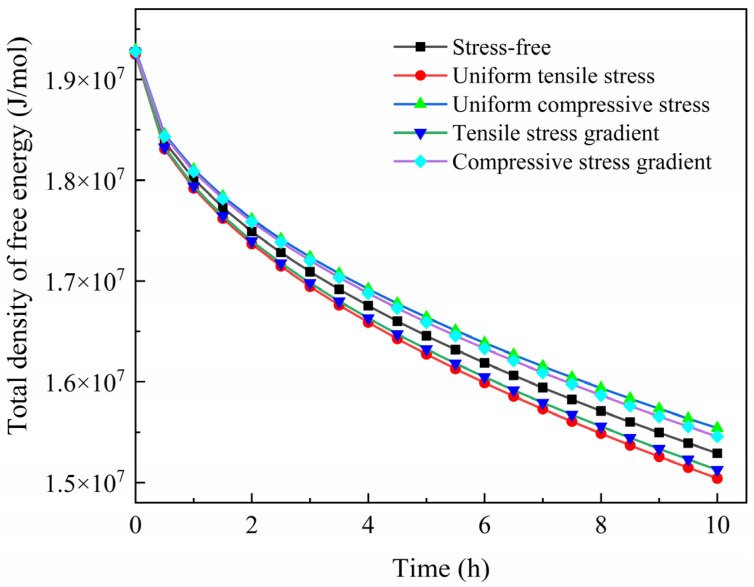
Change in the total density of free energy with diffusion time.

The evolution of the total density of the free energy for the entire system is shown in Figure 11. The total density of free energy is the same at the beginning for the system under different stress states. It is decreased with the diffusion time. The system under uniform tensile stress exhibits the lowest total density of free energy after diffusion. On the contrary, the total density of free energy is highest for the system under uniform compressive stress. Compared with the uniform stress, the work carried out by the gradually decreasing stress is fairly small, which affects the driving force of diffusion slightly.

**Figure 12:**
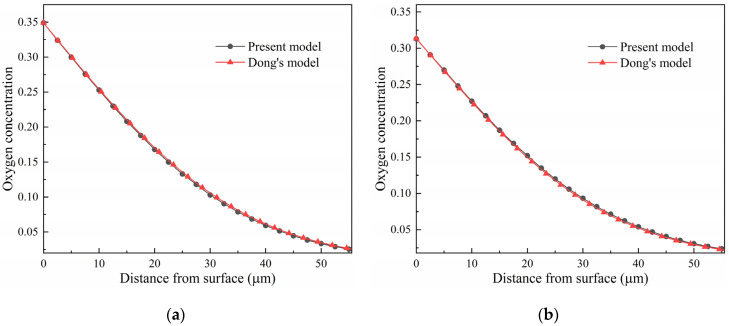
Comparison between the present phase-field model and Dong’s model for oxygen diffusion in pure titanium at 1073 K under different stress: (**a**) 100 MPa, and (**b**) −100 MPa.

The results computed using the present phase-field model indicate that the diffusion behavior is different under different stress states. Quantitative analysis of stress’s effect on atom diffusion is useful for optimizing the processing technology and evaluating the experimental data. As mentioned before, the diffusion behavior under stress can also be described using the modified Fick’s law. Dong and co-authors derived an equation by considering the chemical potential and diffusivity affected by stress to interpret the stress–diffusion coupling effects during oxidation [37]. Using statistical thermodynamics, Yang also derived an analytical expression of diffusivity and the net flux of atoms, which brings out stress’s effects on the diffusion of atoms in a stress field [38]. A comparison of the predicted oxygen concentration distribution in pure titanium between the present phase-field model and Dong’s model is shown in Figure 12. It is demonstrated the results computed using the present phase-field model and Dong’s model are in good agreement.

**Figure 13:**
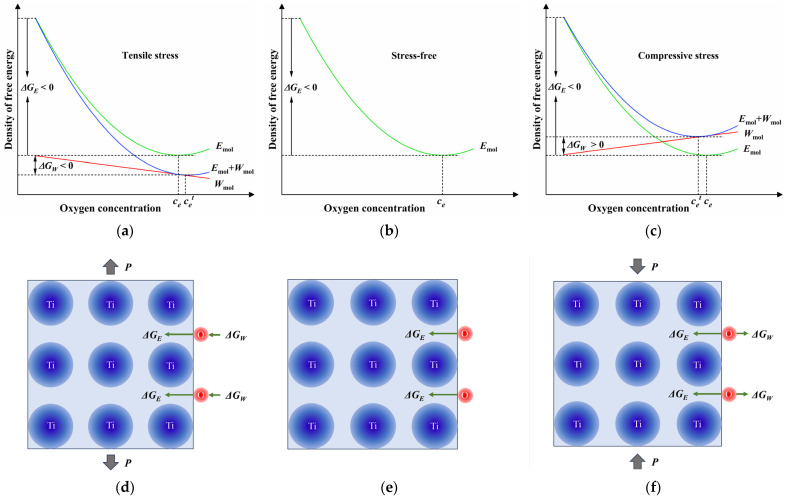
Schematic illustration of the stress’s influence on diffusion: (**a**–**c**) depict the change in free energy with oxygen concentration under different stress states. Correspondingly, (**d**–**f**) illustrate the oxygen diffusion with different driving forces.

Unlike the model derived from Fick’s law, the present phase-field model describes the free energy change resulted by diffusion under stress directly and clearly. The concentration field evolves toward the direction of decreasing the free energy. Based on the phase-field model, a schematic illustration is given to explain the influence of stress on diffusion explicitly, which is shown in Figure 13. For the samples with an initial oxygen concentration lower than the equilibrium concentration $c_{e}$, the oxygen diffusion into the titanium matrix may reduce the chemical energy. This indicates that the oxygen diffusion is driven by chemical energy in this case, as shown in Figure 13b,e. It is evident that the work of the tensile stress can decrease the free energy as the concentration of oxygen is increased from Figure 13a. Therefore, the driving force for oxygen diffusion is a combination of the change in chemical energy $∆G_{E}$ and the work undertaken $∆G_{W}$, as illustrated in Figure 13d. The superposition of $∆G_{E}$ and $∆G_{W}$ promotes oxygen diffusion. The true equilibrium concentration $c_{e}^{t}$ under tensile stress is higher than $c_{e},$ determined by temperature, as shown in Figure 13a. On the other hand, in the case of the samples under compressive stress, partially reduced chemical energy serves as the driving force because $∆G_{W}>0$, as shown in Figure 13c,f. As a result, oxygen diffusion is inhibited under compressive stress. In this case, the true equilibrium concentration $c_{e}^{t}$ is lower than $c_{e}$.

In addition to the uniform stress and stress gradient, the influence of complex stress states, such as time-dependent stress, phase-transformation-induced stress, and inhomogeneous stress resulting from second-phase particles, can also be examined using the present phase-field model. By substituting the parameters in this phase-field model, the diffusion of C and N in titanium and other metals can be modeled. In addition to these practical applications, the model could be extended to the field of oxidation and diffusive phase transformation.

## 4. Conclusions

In this study, the free energy change in a Ti-O system resulting from diffusion under stress states was described in the framework of the phase-field method. A phase-field model was developed to investigate the oxygen diffusion in pure titanium under different stresses. Based on these studies, the following conclusions can be drawn.

(1) The true equilibrium concentration of oxygen in pure titanium under stress has been calculated, and it was found that it is increased with tensile stress and decreased with compressive stress. At a temperature of 873 K, it is increased from 0.309 to 0.353 as the stress increases from −100 MPa to 100 MPa. The influence of stress on the true equilibrium concentration -is reduced by temperature. The difference between the true equilibrium concentration with and without stress becomes small with an increase in temperature.
(2) Tensile stress can facilitate diffusion because the change in the free energy of the Ti-O system induced by the work undertaken on the diffused oxygen atoms is negative. The driving force for diffusion originates from the combined effect of a change in chemical energy and the work undertaken by the tensile stress. The promotion of diffusion is more significant as the tensile stress increases. Compared with the stress-free sample, the oxygen concentration of the pure titanium diffused at 873 K for 10 h was increased by about 7% in the surface layer under 100 MPa of tensile stress.
(3) The free energy change in the system, induced by compressive stress during diffusion, is positive. Hence, the driving force for oxygen diffusion supplied by the decrease in chemical energy is partially counteracted by the work carried out by compressive stress. This is why the diffusion is inhibited by compressive stress and enhanced to a greater extent when increasing the compressive stress. Compared with the stress-free sample, the decrease in the oxygen concentration in the surface layer of titanium diffused at 873 K under a compressive stress of −25 MPa was about 2%. It reached about 7% as the compressive stress increased to −100 MPa.
(4) Compared with uniform stress, there is no obvious difference in the oxygen concentration distribution under stress varying from a high level at the surface to zero at a certain depth. This can be attributed to their similar change in free energy density in the surface layer during diffusion.

## Data Availability

The data are contained within the article.
